# High‐Quality Artery Monitoring and Pathology Imaging Achieved by High‐Performance Synchronous Electrical and Optical Output of Near‐Infrared Organic Photodetector

**DOI:** 10.1002/advs.202203870

**Published:** 2022-11-20

**Authors:** Zeyu He, Xiaoyang Du, Caijun Zheng, Xin Yu, Hui Lin, Silu Tao

**Affiliations:** ^1^ School of Optoelectronic Science and Engineering University of Electronic Science and Technology of China Chengdu 610054 P. R. China

**Keywords:** artery monitoring, biomedicine, near infrared, organic photodetectors, pathology imaging

## Abstract

Near‐infrared organic photodetectors (NIR‐OPDs) are significant technologies in emerging biomedicine applications for uniquely wearable, noninvasive, low‐cost advantages. However, biosignals are weak and changing rapidly so practical biodetection and bioimaging are still challenging for NIR‐OPDs. Herein, high‐performance NIR‐OPDs with synchronous optical output are realized by recombining anode‐injected electrons with photogenerated holes on emitters. Owing to high detection performance of 4.5 × 10^12^ Jones detectivity and 120 kHz −3 dB bandwidth, five arteries are monitored by transmission‐type method and cardiac cycle is analyzed. Importantly, the synchronous optical output is direct emission demonstrating outstanding photon conversion efficiency approaching 20% and luminance signal‐to‐noise ratio over 8000. Consequently, pathology imaging is directly developed without complex readout circuits and arrays from which squamous metaplasia of cervix and carcinoma of large intestine are observed clearly. The NIR‐OPD demonstrates strategies for high‐performance synchronous electrical/optical output and directly imaging. Biomedicine applications implemented here are high level, representing important steps for NIR‐OPDs toward providing clues for clinical diagnosis.

## Graphical abstract

A new concept of near‐infrared organic photodetectors (NIR‐OPDs) that has synchronous electrical and optical output is proposed so that significant biomedicine applications of high‐quality artery monitoring and pathology imaging are both achieved in one OPD. Importantly, the imaging is directly observed by the naked eye, which is largely more convenient than the traditional imaging method.

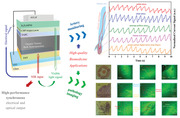

## 1 Introduction

Organic semiconductors, that have designable optical and electronic properties and unique merits of low cost, flexibility, and solution processability, offer a significant platform for the development of optoelectronic devices.^[^ ^1^, ^2^, ^3^, ^4^, ^5^ ^]^ Among them, great emphasis and research are placed on near‐infrared organic photodetectors (NIR‐OPDs), which converts NIR light into electrical signals.^[^ ^6^, ^7^, ^8^ ^]^ One of the principal reasons is that NIR‐OPDs, responding to NIR light that acts on human being tissue, plays a key role for emerging applications of capturing biosignals of human body.^[^ ^8^, ^9^, ^10^ ^]^ More importantly, the applications are promising to be realized in a wearable, light‐weight, low‐cost, low‐radiation, and noninvasive way compared with conventional medical systems, which is of vital significance for better monitoring human‐being health and providing clinical clues of manifestations of diseases.^[^ ^11^, ^12^ ^]^

Recent years, efforts and works for studying and developing NIR‐OPDs are increasing, however, their practical applications on capturing human being biosignals are still facing challenges. First, the biosignals from NIR light that acts on human tissue (especially transmissive light) are generally extremely weak, so that the high performance of detection characteristics is vital for NIR‐OPDs to respond such weak biosignals.^[^ ^13^, ^14^, ^15^ ^]^ Meanwhile, some of the significant biosignals, for example, artery pulse, change rapidly, therefore, it is also essential for NIR‐OPDs to achieve fast speed to respond to biosignals that change rapidly.^[^ ^12^ ^]^ To achieve high detection performance, the bulk heterojunction (BHJ) is preferred as the photoactive layer (PAL) in the OPDs because of its superior photogenerated exciton dissociation efficiency.^[^ ^16^, ^17^ ^]^ Additionally, applying reverse bias is in favor of better dissociation of photogenerated excitons and carriers transport, largely increasing both responsivity and speed to satisfy the demand of capturing weak and rapid biosignals.^[^ ^7^ ^]^ However, due to possible direct contact between acceptor materials and anode, the possibility of injection of electrons from the anode to the lowest unoccupied molecular orbital (LUMO) of the acceptors under reverse bias is rising, which, unfortunately, causes undesirable and serious dark current rise, leading to heavy degradation of detection performance.^[^ ^9^, ^18^, ^19^, ^20^, ^21^ ^]^ This scenario is more severe in NIR‐OPDs due to smaller injection barriers.^[^ ^7^ ^]^ Second, transmission‐type bioimaging via NIR‐OPDs is a significant application in virtue of that the imaging is low radiation and noninvasive. Moreover, this imaging is 3D which means that these bioimages may uniquely contain the internal information including density and thickness of biological tissue.^[^ ^22^ ^]^ However, in addition to the challenge of that biosignals is too weak to be imaged, general dependence on readout circuits and arrays also greatly increases the difficulty of its implementation of bio‐imaging by utilizing NIR‐OPDs.^[^ ^23^, ^24^, ^25^, ^26^ ^]^ Consequently, it is of vital significance to improve the performance of NIR‐OPDs and reduce the complexity and difficulty of bioimaging application implementation, further promoting the development of NIR‐OPD in the exploration of human bio‐signals.

Here, we report a NIR‐OPD that photogenerated electrons from PAL are collected by cathodes, however, photogenerated holes are not collected by anode directly like traditional OPDs, but recombine in emitters with anode‐injected electrons. So that the NIR‐OPD provides a synchronous output of electrical and optical signals and it should be emphasized that the optical signal is in form of direct emission, which greatly facilitates the implementation of imaging applications. Significantly, owing to the sufficient photogenerated carriers provided by PAL of ternary BHJ and the key role of transport and blocking effect played by each functional layer, the electrical and optical signals are both high‐performance in NIR region. For electrical output, an outstanding responsivity of 0.545 A W^‐1^, a high −3 dB bandwidth over 100 kHz and a high detectivity of 4.5 × 10^12^ Jones are achieved. For optical output, wide operation region (power density from 1.68 low to 0.0014 mW cm^‐2^, driving voltages from −5 to −8 V), high luminance signal‐to‐noise ratio over 8000, low imaging energy consumption and excellent photon conversion efficiency approaching 20% are realized. Based on these excellent performance and convenient direct optical output, we carry out two significant applications of capturing human being biosignals. One hand, by utilizing our NIR‐OPD as detectors and employing transmissive type method, we obtain human being's artery signals of five different positions in arm/hand in real‐time. The signals are stable, reliable and consistent with clinical manifestations.^[^ ^27^, ^28^, ^29^ ^]^ Strikingly, as a result of high detection ability and high −3 dB bandwidth, the cardiac cycle monitored by us contains rich information and can be analyzed.^[^ ^30^, ^31^, ^32^, ^33^ ^]^ Meanwhile, owing to the convenient and direct emission of optical output, human pathological imaging is successfully carried out without any readout circuit and array by employing our device, which demonstrates normal tissue morphology, squamous metaplasia in the cervix and tubular adenocarcinoma in large intestine. These results not only provide NIR‐OPDs an efficient way to realize high‐performance synchronous electric and signal output and facilitate the implementation of bio‐imaging, but also raise biomedical applications of NIR‐OPDs to a higher level.

## 2 Results and Discussion

**Figure 1:**
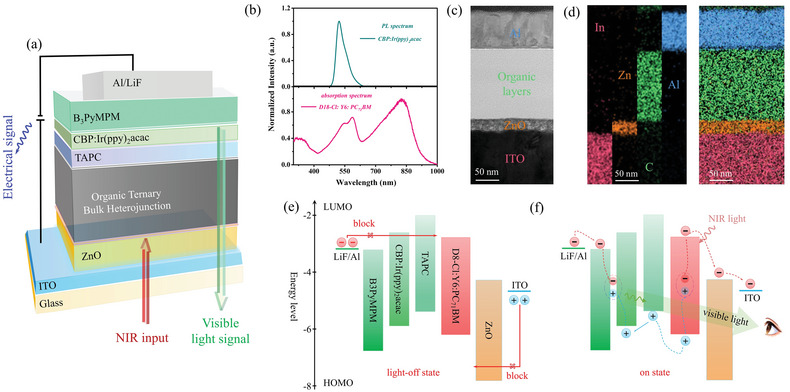
Design and working mechanism of synchronous output of electrical and optical signal featured NIR organic photodetector in this work. a) Schematic of configuration of a synchronous output of electrical and optical signal featured NIR organic photodetector in this work. b) Normalized Absorption spectrum of the PAL and PL spectrum of deposited CBP:Ir(ppy)_2_acac film. c) Cross‐sectional TEM image of the NIR organic photodetector with scale of 50 nm in this work. d) EDS mapping of the NIR organic photodetector and distribution of the main elements (In, Zn, C, and Al) with scale of 50 nm. e) The energy level alignment and working mechanism diagrams of the photodetector in light‐off state. f) The energy level alignment and working mechanism diagrams of the photodetector in on state.

The NIR‐OPD with a synchronous output of electronic and optical signals was fabricated by utilizing spin‐coating method as well as vacuum deposition technology and its configuration was given by a diagram in **Figure** **1**a. Here, from bottom to the top, the functional layers were spin‐coated ZnO/D18‐Cl: Y6: PC_71_BM (in which D18‐Cl is the polymer donor (D), Y6 is the nonfullerene acceptor (A) and PC_71_BM, playing a key role of the third component, respectively and they form an organic ternary bulk heterojunction (T‐BHJ)) and followed by deposited TAPC/CBP:Ir(ppy)_2_acac/B3PyMPM, sequentially (the chemical structures of all organic compounds mentioned above were given in Figure S1, Supporting Information)). ITO and LiF/Al act as transparent cathode and anode at each end of the functional layers, respectively. In the device design, the selection of the ZnO is aiming at extracting photogenerated electrons to ITO for its excellent electron transport properties, and also, ZnO is essential for blocking injected holes because of its deep highest occupied molecular orbital (HOMO) of 7.8 eV.^[^ ^34^ ^]^ In addition, ZnO offers many advantages for the device such as high stability, environmental‐friendly, semi‐transparent and can be easily prepared by sol‐gel strategy.^[^ ^35^, ^36^ ^]^ The mixture of D18‐Cl: Y6: PC_71_BM is an efficient T‐BHJ and constitutes PAL, broadening absorption spectrum range (covering from 300 to 1100 nm exhibited in bottom panel of Figure 1b) compared with each single material absorption capability (shown in Figure S2, Supporting Information). In this T‐BHJ system, the narrow‐bandgap small‐molecule Y6 affords the PAL a strong NIR absorption that peaks around 840 nm, the introducing of third component PC_71_BM beneficially promotes the characteristic of electron transport which is proven in *J*–*V* curve contrast tested by electron‐only devices in Figure S3a (Supporting Information).^[^ ^37^, ^38^ ^]^ It is of vital necessity to consider and investigate that the ternary blending shows a very favorable morphology which brings about excellent performance for the device. That is, one hand, as shown in the atomic force microscopy (AFM) results of Figure S3b (Supporting Information), the film exhibits an even materials distribution with an extremely low root‐mean‐square roughness (RMS) of 0.934 nm. The low RMS is significant thereby indicating that the T‐BHJ gives a well‐contact interface with both ZnO and next layer of the deposited film, which boosts an efficient charge extracting and transporting.^[^ ^39^, ^40^ ^]^ On the other hand, the cross‐section transmission electron microscopy (TEM) images of the T‐BHJ film are provided in Figure S3c (Supporting Information) from which an appropriate phase separation and ample donor/acceptor interfacial areas are observed in the vertical aspects, suggesting that the PAL has superior exciton dissociation and charge transferring to produce high photo‐generated currents for the device, leading high detection performance.^[^ ^41^ ^]^ Further, grazing‐incidence wide‐angle X‐ray scattering (GIWAXS) (in Figure S4d,e, Supporting Information) was measured to study molecular stacking and orientation and the results showed a typical face‐on orientation which is favor of charge transport.^[^ ^9^ ^]^ The selection of deposited films of TAPC/CBP:Ir(ppy)_2_acac/B3PyMPM/LiF/Al is according to typical and high‐performance OLEDs, which is crucial for efficient transport and utilization of photogenerated holes.^[^ ^42^, ^43^, ^44^ ^]^ Further, Figure 1c,d, the images of energy‐dispersive X‐ray spectroscopy (EDS) in a high‐resolution cross‐section TEM of the NIR‐OPD, confirm the structure of the device and from which cross section morphology of each functional layer and each interphase was clearly distinguished (shown in Figure S4, Supporting Information).

Now, we turn to study how the photodetector works and effects of functional layers on device performance through the energy level diagram (shown in Figure 1e,f). When without light radiation as shown in Figure 1e, no exciton generated obviously, meanwhile, HOMO level of 7.80 eV of ZnO plays a key role to effectively prevent holes injected from ITO as well as both hole‐type host CBP and HTL TAPC are crucial to block electrons injected from Al, so that the magnitude of dark current of device may remain almost constant and the device is in light‐off state even along with reverse bias increasing. On the other hand, when light irradiates device (as depicted in Figure 1e), abundant photogenerated excitons were induced and dissociated to free carriers at rich donor/acceptor interfacial areas from T‐BHJ in PAL. Then, the photogenerated electrons are extracted by ZnO and collected to ITO. Meanwhile, as for photogenerated holes, they are transported by HTL and hole‐type host and then reach and are blocked at interface of CBP and B3PyMPM, at which, simultaneously, electrons injected from Al reach as well when the driving voltage is sufficient to overcome the barrier. Ultimately, photogenerated holes and anode‐injected electrons recombine to visible photons in green emitters Ir(ppy)_2_acac. In that way, possible direct contact between acceptor materials and anode is averted and injected electrons from the anode are utilized. Consequently, not only the incident light input can be transformed into output of electrical signal and rise of dark current along with reverse bias increase is suppressed, but also, importantly, the device builds a platform of recombination of photogenerated holes and anode‐injected electrons to photos in emitters, which enables to a synchronous optical signal. It should be emphasized that the signal output is direct emission from emitters, uniquely demonstrating the superiority of imaging applications that is convenient, low‐cost, and independent of readout circuits and arrays.

**Figure 2:**
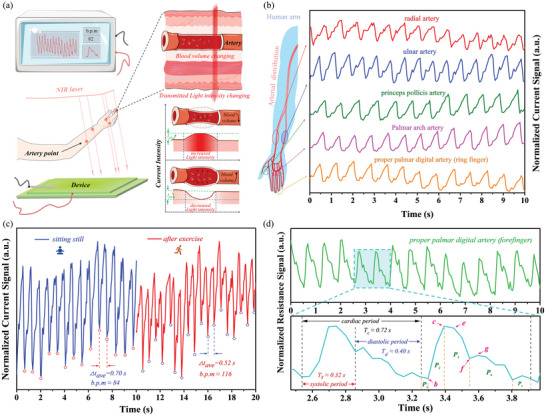
Applications of transmissive type artery pulse monitoring and cardiac cycle analyzing by utilizing our NIR‐OPD. a) Test schematic diagram of transmissive type artery pulse monitoring in real time in this work (the left panel stands test method and the tested results are displayed in real time and the right panel represents test principle). b) The normalized photocurrents artery signals including radial artery, ulnar artery, princeps pollicis artery, palmar arch proper artery, and palmar digital artery. c) The testing results of author's BPM when author keeps calm (blue branch) and is after exercising for 3 min (red branch), respectively. d) The normalized resistance artery signal of palmar digital artery and its enlarged part for analyzing information of cardiac cycle.

By means of the fact that our NIR‐OPDs can convert NIR‐light to electronic output and visual light output simultaneously, our device is considered to be employed in practical applications of bio‐signals capturing. First, artery pulse, as one of the principal signals to assess human being health, can be monitored and analyzed by our device with method of transmissive type (displayed through a schematic diagram in **Figure** **2**a). The test principle can be understood by the right panel of Figure 2a that when NIR light comes through a specific position of human body which includes artery, one part of the light is absorbed by both artery and other tissues. And owing to good transmission capacity of NIR light in human tissues, another part of the light transmits and reaches to our NIR‐OPDs. During the test, blood volume in the artery changes varies over time which causes intensity of the transmitted light changing accordingly. Therefore, the change of photocurrents converted by NIR‐OPDs demonstrates the artery pulse signals. By comparison with the most widely reported pressure method and reflected light type, the transmissive type offers unique superiority that extra pressure comes off and the conditions of tightly keeping a sensor close to skin (for preventing loss of reflected light) is unnecessary, respectively, which may bring people a noninvasive, non‐contact, remote and comfortable monitoring experience.^[^ ^45^, ^46^, ^47^, ^48^ ^]^ However, capturing artery pulse signals in practically by transmissive type facing challenges that on the one hand, locating artery is hard and on the other hand, high performance is vital for NIR‐OPDs to respond transmitted light which is generally extremely weak and the variation speed is fast.

We carried out practical use of transmissive type artery pulse monitoring with 850 nm laser raying author's five arteries on the hand and arm by utilizing our NIR‐OPD as sensor and the results were displayed in a monitor in real‐time (as depicted in Figure 2b). The implementation was carried out in the atmosphere and without help of any external circuits. Interestingly, owing to direct optical output from emission, slight change of luminance on device following artery pulse was observed by our naked eyes and was proven and given by time‐lapse video in supporting video. Visualization of artery pulse is first reported, which is conducive to better locate artery, demonstrating the significant importance of synchronous output of electrical and optical signal in capturing human being biosignals. Figure 2b shows the results of the measurement on five arteries including radial artery, ulnar artery, princeps pollicis artery, palmar arch proper artery, and palmar digital artery. The pulse signals of radial artery, ulnar artery, princeps pollicis artery, palmar arch proper artery monitored by NIR‐OPDs are first reported. Normalized tested current signals on five arteries all exhibited similar and stable time‐dependent wave shapes that contains wave peak, wave trough, and some side peaks. The different offsets of the side peak may depend on the distance between artery and heart. The peak and trough correspond to the time that blood volume is minimum and maximum in artery during a cardiac cycle, which can calculate the human being beats per minute (BPM). Therefore, we verified and tested author's BPM when author kept calm and is after exercising for 3 min (exhibited in Figure 2c) and the BPMs in our tests are 84 and 108, respectively, which tally well with typically physiology characteristics, demonstrating the rationality and correctness of the experiment.^[^ ^27^, ^28^ ^]^ To further analyze the pulse signals, we obtained resistance as function of time shown in Figure 2d, which shows highly consistent with the pulse signal obtained in clinic.^[^ ^29^, ^30^, ^31^, ^32^, ^33^ ^]^ Two enlarged groups of signals (in the bottom panel of Figure 2d) provided rich and detailed signals that systolic and diastolic periods (0.32 s and 0.40 s respectively) in a cardiac cycle can be clearly divided. Additionally, dicrotic notch at "f" point, ascending branch of P_1_, the descent branch from P_2_ to P_5_ and other information were captured clearly (More explanation was given in Figure S5, Supporting Information).^[^ ^29^, ^30^, ^31^, ^32^, ^33^ ^]^ The information of cardiac cycle measured by our device provides the potentiality of assessment and clues for clinical trials such as pulse rate, arrhythmia, and myocardial contractility, etc.^[^ ^30^, ^31^, ^32^, ^33^ ^]^ These results are novel and reach a high level which coincides with and is comparable to clinical invasive artery detection, demonstrating the potential of our NIR‐OPDs in transmissive‐type artery pulse monitoring and analysis.

**Figure 3:**
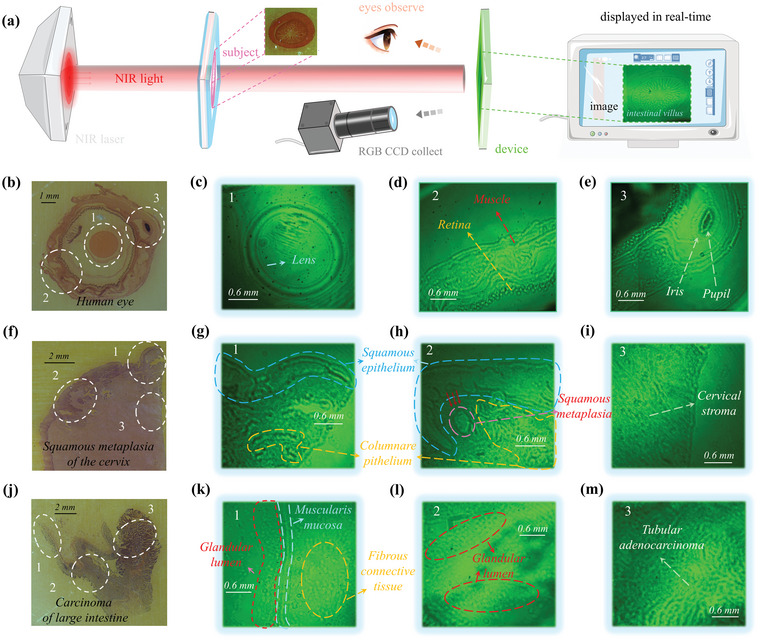
Bioimaging applications of the NIR‐OPD. a) Schematic diagram of bio‐imaging process and method by utilizing our NIR‐OPR and imaging results of small intestine (on the right of the annotation “image”). b) Images of transection of human eye sample directly collected by RGB CCD and c–e) imaging under 850 nm NIR laser. f) Images of squamous metaplasia of cervix sample directly collected by RGB CCD and g–i) imaging under 850 nm NIR laser. j) Images of carcinoma of large intestine sample directly collected by RGB CCD and k–m) imaging under 850 nm NIR laser.

We then sought to further assess the applications of that the device directly converts NIR‐light to visual light output. Transmissive type bioimaging by employing NIR‐PDs gains unique advantages including noninvasive and tiny damage to tissue, and also, the images are capable of not only showing shape and edge of biotissues, but also novelty and significantly giving information of penetration depth variation, which has great potential to provide clues for the future diagnosis of human pathology.^[^ ^22^ ^]^ However, array and readout circuit are often required for practical imaging applications which greatly increases complexity the difficulty.^[^ ^23^, ^24^, ^25^, ^26^ ^]^ Additionally, in consideration of that fine structure in human tissue, it is required for NIR‐PDs to distinguish small transmitted light intensity difference and provide high contrast in image. So, the real imaging of human tissue and pathology by using NIR‐OPDs has not been realized and reported yet. In virtue of direct emission of emitter, imaging application by our device is array free and without readout circuit. Therefore, some subjects were placed between an 850 nm NIR laser and our device for carrying out transmissive‐type bioimaging applications (The imaging diagram is shown in **Figure** **3**a and the actual experiment of imaging is displayed in Figure S6, Supporting Information). The imaging results are on the device and can be observed by naked eye and in order to readily observe images, the image data are collected by a red‐green‐blue (RGB) CCD sensor. Although in our device, the direct emission from emission layer allows the imaging to be theoretically pixel free because the emission is from emitters. However, the actual size of resolution of the images we give collected by RGB‐CCD is governed by the resolution of RGB‐CCD. Consequently, the resolution was tested and the result here was 12.5 line mm^‐1^ (shown in Figure S7a, Supporting Information). This resolution is high enough to analyze the edge and morphology of biological tissue.

Now we perform bio‐imaging and as shown in Figure S7b–d (Supporting Information), specimens of locust wing slice and fly wing were imaged extremely clearly by imaging system that the profile, and texture of the two wings was distinguished and viewed vividly. Additionally, cerebellar cortical layer imaging showed clear outline. More importantly, the image obtained by our device demonstrates more penetration depth variation information of housefly mouth compared with the image obtained by RGB‐CCD directly (Figure S7e, Supporting Information), which proves the high importance and novelty of the proposed device. Then, a further attempt was developed on human eyes. As shown in Figure 3b, the transection of human eye was imaged by our device that part of fine structure of eyes including the lens (Figure 3c), muscle distribution and retina (Figure 3d), pupil and iris (Figure 3e) were found, indicating that our NIR‐OPDs and bioimaging system has the potential to image the fine structure of human body. In the right of Figure 3a, the fine structure of intestinal villus was clearly observed. Furthermore, more experiments were developed to promote human pathology imaging. And the as shown in Figure 3f, a cervical sample and its imaging results were given. Fibrous stroma was shown in Figure 3i and squamous epithelium and columnar epithelium were distinguished in Figure 3g,h as a result of their different shapes and densities. Importantly, squamous metaplasia was clearly observed in the cervical shown in Figure 3h.^[^ ^49^ ^]^ Figure 3d further shows a group of images of the pathology of the cancerous large intestine tissue sample, from which muscularis mucosa (appear as a line) and fibrous connective tissue inside (relatively loose) was displayed (Figure 3k). Also, the small gland cavity of normal intestinal mucosal glands presents a regular ring‐like shape (Figure 3l) with a relatively even distribution. Interestingly, due to irregular disorder and densely packed cells, the image of the cancerous part (Figure 3m) shows obvious cellular atypia of tubular‐shaped and weaker emission compared with normal one. The imaging results, therefore, demonstrate that squamous metaplasia in cervix, normal and cancerous tissues in the large intestine can be successfully imaged, observed, and distinguished by employing our device. Successfully realizing transmission‐type high‐quality human pathological imaging by utilizing OPDs is the first reported, which is an extremely significant highlight and indicate that our device may offer a potential platform for health monitoring and pathologic diagnosis with noninvasive method.

The practical application on bioimaging has demonstrated that our high‐performance NIR‐OPD is a great candidate that can directly image without complex readout circuits and arrays. And more significantly, this technology establishes a more effective human–machine dialogue, that is, our NIR‐OPD achieves synergies mode which converts the information of the detecting target to be digitization by electrical output and visualization by optical output, simultaneously. The synergies between electrical output and optical output demonstrate tremendous advantage and potential, providing an immersive interactive experience with both naked‐eye readable and high electronic precision for future applications such as human–machine interface, virtual reality, and rescue, and information interaction.

**Figure 4:**
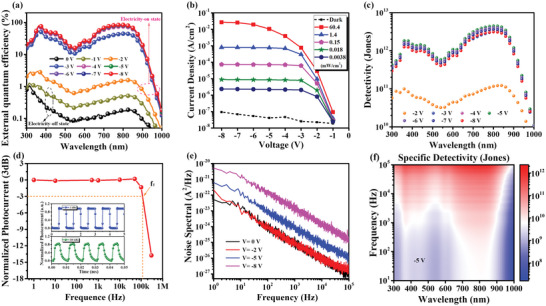
The detection performance of the NIR‐OPD. a) The EQE spectrum of the NIR‐OPD at different reverse bias. b) Current density as function of reverse bias of the NIR‐OPD in the dark and under radiation of 808 nm laser with different input power density. c) The detectivity as function of wavelength with different reverse bias. d) Normalized photocurrents as function of modulating frequency and normalized photocurrents of the NIR‐OPD modulated by frequency of 1 kHz and 100 kHz (top panel and bottom panel in the insert Figure, respectively). e) Frequency‐dependent noise spectrum of the NIR‐OPD with different reverse bias applying. f) Specific detectivity mapping (as a function of both frequency and incident light wavelength) at reverse bias of −5 V.

Next, we start to analyze the performance of the output of electrical signal and optical signal of the optimized NIR‐OPD from which to clarify the reasons of application implementations of biosignal capture realized by our device can reach such a high level. First, we explore in detail of characteristics of output of electrical signal. The EQE as function of wavelength under different driving voltages was prepared in **Figure** **4**a. Here, when there was no bias voltage or −1 V was applied, evidently, electrons hardly injects and few electrons are able to overcome the barrier from cathode, so that this electricity‐off state of device makes the values of EQE close to 0 even if photogenerated carriers produced. And when higher reverse bias, which is sufficient to overcome barriers, was applied, the device is in electricity‐on state and it can be seen that the EQE values climbed with increasing of voltages. The climb of EQE can be understood that the photogenerated holes from PAL, recombine to photons depending on the quantity of electrons provided by reverse bias. The EQEs reach extremely high values of 80.9% to 84.4% at bias of −5 to −8 V under 800 nm, which are higher than both the standard silicon detector and control device (the control device is a traditional OPD with same PAL shown in Figure S8a, Supporting Information and results was given in Figure S8b, Supporting Information). High bias is a principal contributing factor to high EQEs, which further promote generation of exciton and transport of carriers. The responsivity (*R*) was further analyzed by equation of $R=\mathrm{EQE}\cdot e/E_{\mathrm{ph}}$, where *e* and *E*_ph_ represent the elemental charge and photon energy, respectively. As expected, a very high value of R reaching to 0.545 A/W was obtained (more details are shown in Figure S9a, Supporting Information), which is one of the highest values among reported NIR photodetectors.^[^ ^13^ ^]^ Excellent responsivity of the NIR‐OPDs ensures well responding to weak arterial signal change. To better demonstrate photoresponse of the device, we tested the photocurrents as function of input powers under different wavelengths in NIR region. The results are exhibited in Figure S9b (Supporting Information) and we observed that the data has linear trend in log coordinate as reverse bias increasing. It should be noted that, importantly, as input power increasing higher, the value of saturation photocurrent increases with the increase of reverse bias, which is consistent with climb of EQE mentioned above that photogenerated holes that can recombine to photons depend on the quantity of electrons provided by reverse bias under a specific incident light intensity. The wide linear trend can be fitted well by equation of $I_{\mathrm{ph}}=\alpha P_{\mathrm{in}}^{\beta }$ (*I*_ph_ and *P*_in_ stand the photocurrent density and input power density (*P*_in_), respectively) from which fitting curve shows *β* of 0.968 according well with test data and a linear dynamic range (LDR) of 82.5 dB is found. It is vital of necessity to state that the tested LDR is limited by our test conditions of limited input power and should have been higher because no saturation shows at lower power density, clearly. So that we measured dark current of device to further investigate detection performance and as shown in Figure 4b, the dark currents were below 10^−8^ A in wide reverse bias from 0 high to −8 V and the highest on/off ratio of 2.9 × 10^5^ was obtained (shown in Figure S9d, Supporting Information), which effectively prevents the annihilation of weak pulse signal. The low rise of dark current along with reverse bias increase attributes to that, superior to traditional OPDs, in our device CBP, TAPC are crucial to block electrons injected from Al and the possible direct contact between acceptor materials and anode is averted for the introduction of functional layers between anode and ATL. Interestingly, the operation range high to −8 V and maintaining low dark current is rare and rarely reported among NIR‐OPDs.^[^ ^18^, ^19^, ^20^, ^21^ ^]^

With *R* and dark current obtained, then, we calculated detectivity (D*), a more convincing expression of detection performance, by employing formula: $D^{*}=R\sqrt{A}/\sqrt{2qI_{d}}$, where *A* and *I*_d_ stand effective area of device and dark current, respectively. The D* as function of both reverse bias and wavelength was shown in Figure 4c and a high D* of 4.5×10^12^ Jones achieved at 810 nm under −5 V. In addition, frequency‐dependent specific D* was characterized as well in order to more completely probe into the detection performance of our device under frequency modulation. First, time‐resolved normalized photocurrents (NPs) with different operation frequency were tested. As shown in Figure 4d, the 850 nm laser on‐off switching modulated NPs with frequency (*f*) of 1 kHz and 100 kHz, respectively. When modulated by laser of 1 kHz, the device showed a NP with intensity of 1, which demonstrated that the device responds a complete signal. While the device was modulated by laser of 100 kHz, the NP intensity still remains at 85.7% intensity. Further, 3 dB bandwidth (*f*_T_), representing cut‐off of dependent on modulation frequency (when complete NPs intensity dips to 70.7%), is given in Figure 4d. It is obvious that *f*_T_ is more than 100 kHz, which is comparable with reported high‐speed NIR‐OPDs and shows that our device has feasibility in application scenarios of high‐frequency optical modulation. The device has high *f*_T_ owing to high reverse bias which is in favor of carrier transport and fast recombining to photons. The wide modulation frequency region is of great significance to prevent distortion of rapidly changing artery signals. Based on wide frequency modulation range from 1 to 100 kHz, so that, noise spectral measurement as function of frequency at different reverse bias was tested in Figure 4e intending to analyze total noise current of the device and prepare to calculate specific D*. As result shown in Figure 4e, noise spectrum displays a typical 1/*f* dependent trend from 1 to 10^4^ Hz and flicker noise may be the major factor that limits detection performance in low‐frequency region. Meanwhile, growth of *S_n_* is observed when reverse bias is applied and increases, which is identical to previous results of dark current measurement. Therefore, specific D* was further calculated by using formula of $D^{*}=R\sqrt{A}/S_{n}$. Figure 2d demonstrates a mapping both wavelength and modulating frequency dependent of specific *D* ^*^ that specific D* over 10^12^ Jones in NIR region was also obtained.

**Figure 5:**
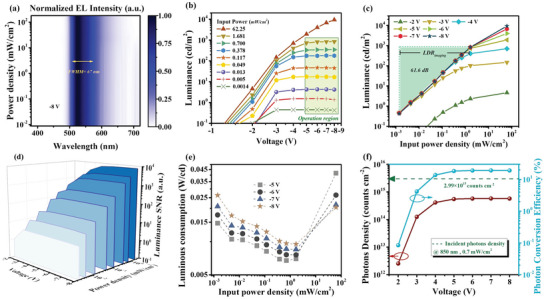
Characteristics and performance of optical output of the NIR‐OPD. a) The normalized EL spectrum mapping a function of both wavelength and input power density at bias of 8 V under 850 nm light radiation. b) The luminance–voltage curves under different input‐power‐density 850 nm light radiation. c) The luminance as function of input power density with different bias applying and imaging LDR. d) The luminance signal‐noise‐ratio (SNR) and contrast as function of both bias and input power density. e) Luminance consumption as function of both bias and input power density. f) The photos density as function of bias and photon conversion efficiency curves.

We have explored and studied above‐mentioned performance of electrical output of our NIR‐OPDs that an excellent *R* of 0.545 A W^‐1^, a high D* of 4.5×10^12^ Jones and wide frequency modulated range over 100 kHz −3 dB are achieved. The outstanding electrical output is the guarantee of obtaining high‐level arterial signals and analyzing cardiac cycle. Then, we further start to investigate the characteristics of optical output. Here, the electroluminescence (EL) spectrum of optical signal was characterized. Given normalized EL intensity as a function of voltage (1.5 mW cm^‐2^ 850 nm radiation) in Figure S10a (Supporting Information), spectra peaking around 525 nm with full width half maximum (FWHM) of 67 nm were obtained. Importantly, the only peak of EL spectra is from emitter Ir(ppy)_2_acac demonstrating that energy of exciton which is recombined by photogenerated holes and anode‐injected electrons, transferred to emitters by effective Förster energy transfer, stating high‐efficiency utilization of photogenerated holes. Meanwhile, normalized EL intensity shows independent of input power density according to EL spectrum mapping provided in **Figure** **5**a, which ensures the spectral stability of imaging applications. We further explored and researched performance of optical signal excited by 850 nm, which is regarded as the significant wavelength of tissue transparency window and had been used for bio‐imaging in this work. First, voltage‐dependent luminance curves at different input power density were performed in Figure 5b. Here, low input power density of 0.7 mW cm^‐2^ and extremely low driving voltage of −1.35 V were sufficient to drive the device to emit with luminance of 0.1 cd m^‐2^. The low driving voltages are benefited from device design that we make and limit emission region at interface of ETL and hole‐type host rather than electrons further overcoming barrier of the hole‐type host for injecting into hole‐type host from ETL. Moreover, luminance was gradually saturated as increase of bias and the saturation values increased following input power density. This saturation accords closely with the previous discussion on photocurrent saturation. The minimum input power density which is able to convert to emission is 1.4 µW cm^‐2^, proving that the device has superior capability of imaging small biological details. It is essential to point out that from 0.0014 to 1.68 mW cm^‐2^, the optical output gives stable luminance with bias changing from −5 to −8 V. To understand the significance of stable operation region for bio‐imaging, we prepared input power density dependent luminance curves at different voltage exhibited in Figure 5c. The operation region demonstrated good linear relationship between luminance and input power density and this imaging linear dynamic range can be defined and calculated as equation of ${\mathrm{LDR}}_{\mathrm{imaging}}=20\log\left(\frac{L_{\max}}{L_{\min}}\right)$, where *L*_max_ and *L*_min_ stands the maximum and minimum luminance in the operation region, respectively. The imaging LDR was calculated to 61.6 dB, indicating that the device provides bio‐imaging applications wide liner region in which transmitted NIR light from an organism can be linearly transformed into distinguishable luminance information of organism (including profile, shape, and density). It should be noted that the output of the optical signal may be limited by luminance noise (the luminance when without NIR light radiation), which may lead to a decline in low‐brightness imaging quality. Therefore, luminance noise in the dark was measured and was all below 0.1 cd cm^‐2^ (the accurate value is missed due to the limited resolution of our brightness sensor) at bias region from 0 to −8 V. The very low luminance noise mainly attributes to the blocking effect of each functional layer mentioned before. And the luminance signal‐noise‐ratio (luminance SNR) was obtained in Figure 5d from which the highest luminance SNR, or can be understood as contrast, was over 8000:1. The high contrast is crucial for obtaining high‐quality bio‐imaging. Additionally, luminance SNR over 4.57 was realized in an extremely low input power density of 1.4 µW cm^‐2^ in the operation region, stating the advantages of weak light imaging by using our device. Furthermore, we researched and assessed the energy consumption of imaging. Considering that the signal output is in form of luminance of emission and the input energy originates from power of driving voltages (*P*_v_ = *IV*) and NIR light radiation (*P*_i_), so that the luminance consumption here was defined and expressed as (*P*_v_ + *P*_i_)/*L*, where *L* is emission luminance of optical output. As displayed in Figure 5e, lowest luminance consumption of 0.0067 W cd^‐1^, representing that consuming power low to 0.0067 W can drive a device with unit luminance intensity. And in comparison, the obviously higher consumption was found in the saturation region due to absorption saturation of PAL for high power density light, which is consistent with the photocurrent and brightness saturation discussed before. The low luminance consumption in operation region principal attributes to well‐designed device structure of efficient photogenerated exciton generation/transport and high‐performance luminescence mechanism of the deposited film system (shown in Figure S10b,c, Supporting Information). As shown in Figure S10c (Supporting Information), it can be seen that our device shows high optical EQE, that the high values over 28.4% are achieved in bias from −5 to −8 V. When we consider responsivity of optical signal in our device, the inputs are NIR light as well as bias, and the output is emission from emission layer. Consequently, the responsiveness of optical signal is equal to the utilization of incident NIR light to optical output (as function of bias). We strictly define this as the ratio of the visible photons density of emission (*D*_eph_) to the photons density of incident NIR radiation (*D*_iph_), which can be performed as photon conversion efficiency (PhCE) and is calculated by formula of PhCE = *D*_eph_ /*D*_iph_. In stable imaging region, input power density of 0.7 mW cm^‐2^, which is calculated as equivalent to incident photons density (*D*_iph_) of 2.99×10^15^ counts cm^−2^ in unit time by formula of *D*_iph_ = *P*_in_ /*hν* (where *h* stands Planck constant and *ν* stands photon frequency), was applied on our device. As revealed in Figure 5f, the photons density of emission (*D*_eph_) in unit time, calculated by equation of $D_{\mathrm{eph}}=\frac{\int J_{\mathrm{ph}}\lambda }{R(\lambda )hc}\;d\lambda$, where *J*_ph_ and *R(λ)* are photocurrents density and responsivity. The increases of *D*_eph_ from 2.54 × 10^12^ to 5.73 × 10^14^ counts cm^−2^ as bias increasing from −2 to −8 V because bias promotes carrier separation and extraction. The PhCE was calculated approaching 20%, demonstrating the high performance and low power consumption during imaging by utilizing our NIR‐OPD. And according to the definition of electrical signal, we define detectivity value for the optical signal as formula of $D_{\mathrm{LIGHT}}^{*}=\frac{L_{\mathrm{light}}}{P_{\mathrm{in}}\cdot L_{\mathrm{dark}}}$ and the results are shown in Figure S10d (Supporting Information). It can be seen that the device shows high *D* ^*^_LIGHT_ in the operation region from −5 to −8 V with values of 5 × 10^6^ cm^2^ W^‐1^. The high *D* ^*^_LIGHT_ values in operation region are consistent with previous discussions on consuming power and PhCE, which demonstrates the high‐performance optical signals of our device in the operation region.

## 3 Conclusion

In this paper, we demonstrate a NIR‐OPD in which photogenerated electrons are transported by ZnO to cathode of ITO and photogenerated holes recombine with injected electrons at green emitters, emitting green light, ultimately. Consequently, the device provides a synchronous output of electrical and optical signals. Importantly, owing careful selection of PAL which efficiently generates and dissociates photogenerated excitons and each functional layer which transports and utilizes photogenerated electrons and holes with high efficiency, the electrical and optical signals of the device are both high performance. For electrical output, an outstanding *R* of 0.545 A W^‐1^, a high D* of 4.5 × 10^12^ Jones and wide frequency modulated range over 100 kHz are achieved. And for optical output, high luminance signal‐to‐noise ratio over 8000, minimum converting‐emission input power density of 1.4 µW cm^‐2^ and excellent photon conversion efficiency approaching 20% are obtained. The above‐mentioned high performance and direct output of optical signal are vital and advantageous for our NIR‐OPD to be used in applications of biosignals capture. Therefore, five arteries including radial artery, ulnar artery, princeps pollicis artery, palmar arch proper artery, and palmar digital artery, located by flicker of optical signal output, are monitored by our device from which BPM, cardiac cycle (including diastolic and systolic) and dicrotic notch are observed and analyzed. In addition, owing to direct emission of optical output, human pathological imaging is successfully carried out without any readout circuit and array by employing our device, which demonstrates normal tissue morphology, squamous metaplasia in cervix and tubular adenocarcinoma in large intestine. The artery signals we got are novel and the signals can be compared with clinical invasive pulse monitoring and also human pathological imaging realized by OPDs are first reported. These results demonstrate that our device not only provides a novel design for NIR‐OPDs to achieve high performance both in electrical and optical output, but also the applications we realized have taken capturing biosignals by NIR‐OPDs a higher level.

## 4 Experimental Section

### Device Fabrication and Characterization

The ITO substrate was washed by the washing detergent and dried in the oven at 120 °C for 2 h. Then, ZnO film was prepared on the ultrasonically cleaned and UV‐O_3_ treated ITO substrate with applying to sol‐gel strategy, which dissolved 31 mg of ethanolamine and 110 mg of zinc acetate dihydrate (Zn(CH_3_COO)_2_·2H_2_O) in 1 mL of 2‐methoxyethanol and stirred the solution at least 10 h, after that the solution was spin‐coated on ITO and annealed in the air with 200 °C for 1.5 h form ZnO film. PAL film was made by utilizing a solution process, that is, first, D18‐Cl, Y6, and PC_71_BM were mixed (1:1.5:0.1, w/w) and dissolved in chloroform solution (total concentration of 10 mg mL^‐1^) and then were spin‐coated onto prepared ZnO film with chloroform solvent annealing treating for 5 min in the glove box. Then, the sample was transferred into the vacuum deposition system and the air pressure in the chamber is lower than 10^−5^ Pa. And then, the optimized thickness of organic layers of TAPC, emission layer, and B3 PyMPM are 30, 30, and 50 nm, respectively. The best weight ratio of CBP and Ir(ppy)_2_acac in the emission layer is 93:7. All of those organic layers were deposited with the speed of 1 Å s^‐1^. And then the LiF and Al were with thickness of 0.8 nm and 100 nm and their deposited speed were 0.1 and 5 Å s^‐1^, respectively. The effective area of the device is 0.1 cm^2^.

### Measurement Methods

The absorption spectra were measured by Hitachi U‐3010 UV–VS. PL spectrum was recorded by F‐4600 spectrophotometer. Atomic force microscope (AFM, Asylum Research AFM system (MFP‐3D‐BIO)) was performed in “Ceshigo Research Service, www.ceshigo.com”. GIXRD was tested by X‐ray diffractometer (EMPYREAN). Energy‐dispersive X‐ray spectroscopy (EDS) in cross‐sectional high‐resolution TEM was investigated by Helios NanoLab 600i. The sample was radiated at 10.0 keV X‐ray with an incident angle of 0.10°. Grazing‐incidence wide‐angle X‐ray scattering (GIWAXS) measurements were carried out at the BL02U2 station at Shanghai Synchrotron. The incident X‐ray photon energy was 10 keV and the angle of incidence was 0.08°. External quantum efficiency (EQE) properties were performed with a Keithley 2000 source meter instrument and a QEX10 Quantum Efficiency Measurement System. The electrical characteristics of electron‐only device were measured by Keithley 2400. All electrical and photoresponse characteristics of devices were measured by a Keithley 2400 source meter analyzer and PDA analyzer. The optical characteristics of the devices were recorded by a Spectrascan PR655 photometer in the air. The frequency‐dependent noise spectrum, highly time‐resolved photoresponse characteristics (performance of response speed) of the device were tested by PDA analyzer with probe station. For biomedical image, the imaging laser intensity and driving voltages are in the operation region, the distance between the subjects and NIR‐OPDs is exceeding 5 cm.

## Conflict of Interest

The authors declare no conflict of interest.

## Author Contributions

S.T. and X.D. conceived and supervised the project. Z.H. prepared the device and performed the measurements and characterizations. Z.H. and X.D. performed the data analysis. Z.H. wrote the original draft. All authors discussed the results and commented on the manuscript.

## Supporting information

Supporting Information

Supplemental Video 1

## Data Availability

The data that support the findings of this study are available from the corresponding author upon reasonable request.
