# Supplementary material for: High‐Quality Artery Monitoring and Pathology Imaging Achieved by High‐Performance Synchronous Electrical and Optical Output of Near‐Infrared Organic Photodetector
Source: Adv Sci (Weinh). 2022 Nov 20;10(2):2203870. doi: 10.1002/advs.202203870 (PMC9839857; doi:10.1002/advs.202203870)
Supplement: Supplementary file 1 — Supporting Information [file ADVS-10-2203870-s002.pdf]

Supplementary Information

High-Quality Artery Monitoring and Pathology Imaging Achieved by High-Performance Synchronous Electrical and Optical Output of Near-Infrared Organic Photodetector

Zeyu He, Xiaoyang Du, Caijun Zheng, Xin Yu, Hui Lin and Silu Tao**

Dr. Z. He, Dr. X. Du, Prof. C. Zheng, Dr. X. Yu, Dr. H. Lin and Prof. S. Tao
School of Optoelectronic Science and Engineering, University of Electronic Science and Technology of China, Chengdu 610054, China
E-mail: xiaoyangdu@uestc.edu.cn, silutao@uestc.edu.cn,

I. Supplementary Methods

1. Materials Information

The donor (D18-Cl) and acceptors (Y6) and the third component ($PC_{71}BM$) were purchased from Solarmer Materials Inc. (Beijing). Di-[4-(N,N-di-p-tolyl-amino)-phenyl]cyclohexane (TAPC), 4,4'-Bis(carbazole-9-yl)biphenyl (CBP), Bis(2-phenylpyridine)(acetylacetonate) iridium(III) (Ir(ppy)2acac), 4,6-Bis(3,5-di(pyridin-3-yl)phenyl)-2-methylpyrimidine (B3PyMPM) and LiF were purchased from Luminescence Technology Corp. 1-chloronaphthalene (CN), Zinc acetate dihydrate, ethanolamine, MoO_3 and 2-methoxyethanol were purchased from Sigma-Aldrich Inc. All reagents and solvents were used directly as received. The biological samples or sections were purchased from Suzhou Shenying Optical Co. LTD and Xinxiang Hongye edu. Instrument Co., All reagents and solvents were used directly as received.

2. Electron-Only Device Fabrication

We fabricated the ternary BHJ based or binary BHJ based electron-only devices to demonstrate the significant importance of third component $PC_{71}BM$ as improvement of electron-transport ability, where their structure was ITO/ZnO (30 nm)/D18-Cl:Y6:$PC_{71}BM$ (1:1.5:0.1 wt%) (70 nm)/ LiF (1 nm)/ Au (100 nm) or ITO/ZnO (30 nm)/D18-Cl:Y6 (1:1.6 wt%) (70 nm)/ LiF (1 nm)/ Au (100 nm), respectively. The ZnO was spin-coated on the cleaned ITO substrates by using sol-gel strategy with speed of 5000 rpm and then were transferred to a hot plate immediately for thermal annealing (200 °C, 1h). The BHJs were prepared on ZnO in the glove box with a speed of 3600 rpm by utilizing solution process (mixture blend solution of BHJs were dissolved in CF) with concentration of 10 mg/ml). After chloroform solvent annealing for 5 min, they were transferred to a vacuum evaporation chamber (10^{-5} Pa) and LiF/Au were sequentially deposited on the BHJs

II. Supplementary Fig. S1 to 10

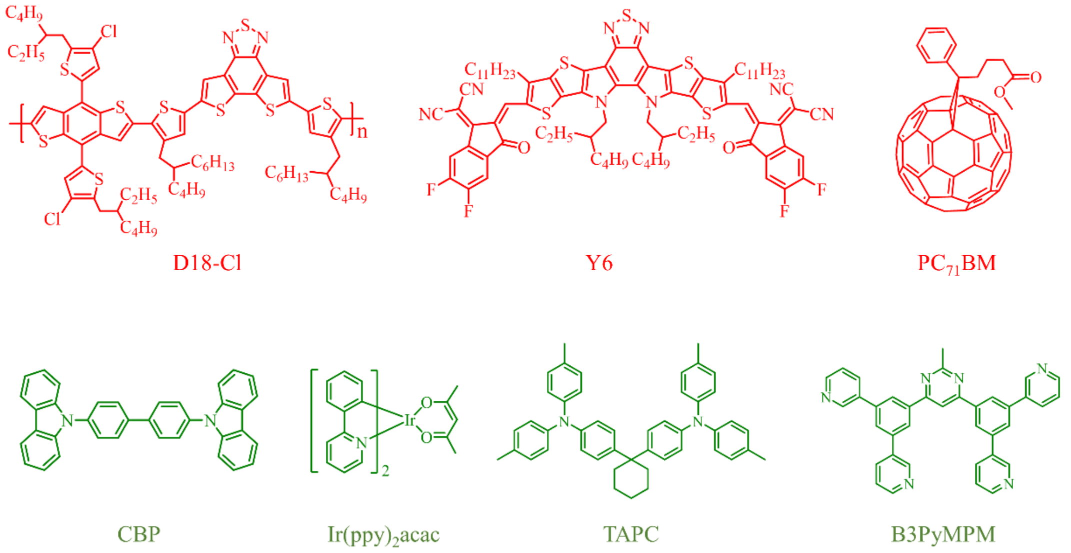

Fig. S1. The chemical structures of all organic compounds used in this work.

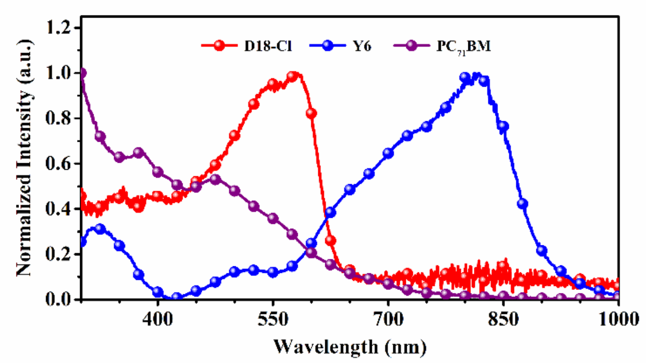

Fig. S2. The absorption spectrum of each single material in PAL.

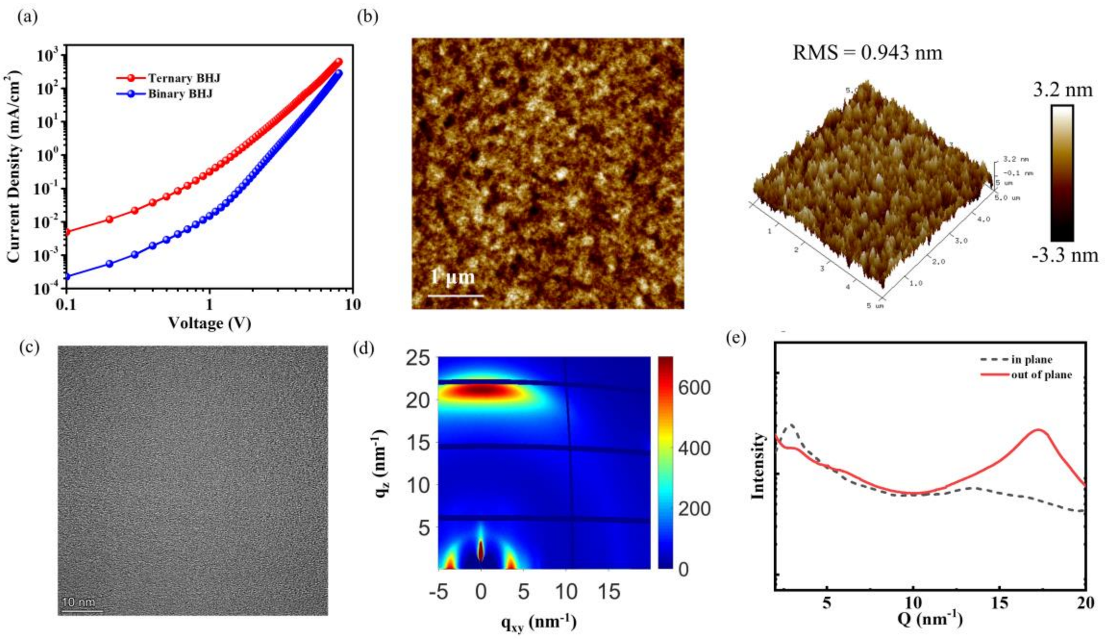

Fig. S3. Contrast of electron-transport characteristic and morphology analysis of organic ternary bulk heterojunction film. (a) J-V curves of binary and ternary BHJ based electron-only devices. (b) Two-dimension (left side) and three-dimensional (right side) AFM images of the solution-processed OT-BHJ used as PAL in this work with scale of 1 μm, RMS=0.943 nm. (c) Cross-section TEM image of the NIR organic photodetector with scale of 100 nm in this work. (d). 2D GIWAXS images of the PAL films blend. e) Out-of-plane and in-plane line-cut profiles for the film of the PAL.

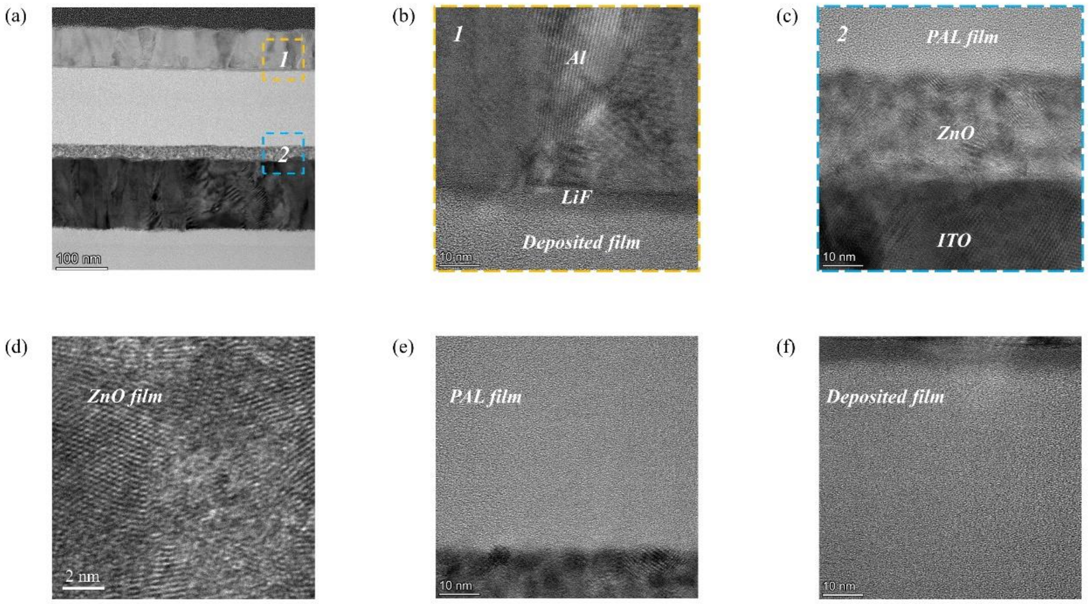

Fig. S4. Morphology characteristics of Cross-section TEM images. (a) Cross-section TEM images of the NIR-OPDs with the scale of 100 nm. (b) Enlarged cross-sectional morphology of Al/LiF, deposited film and their interface with scale of 10 nm. (c) Enlarged cross-section morphology of ITO, ZnO and PAL layer and their interfaces with scale of 10 nm. (d) High-resolution and enlarged cross-sectional morphology of ZnO with scale of 2 nm. (e) High-resolution and enlarged cross-sectional morphology of PAL layer with scale of 10 nm. (f) High-resolution and enlarged cross-sectional morphology of deposited film with scale of 10 nm.

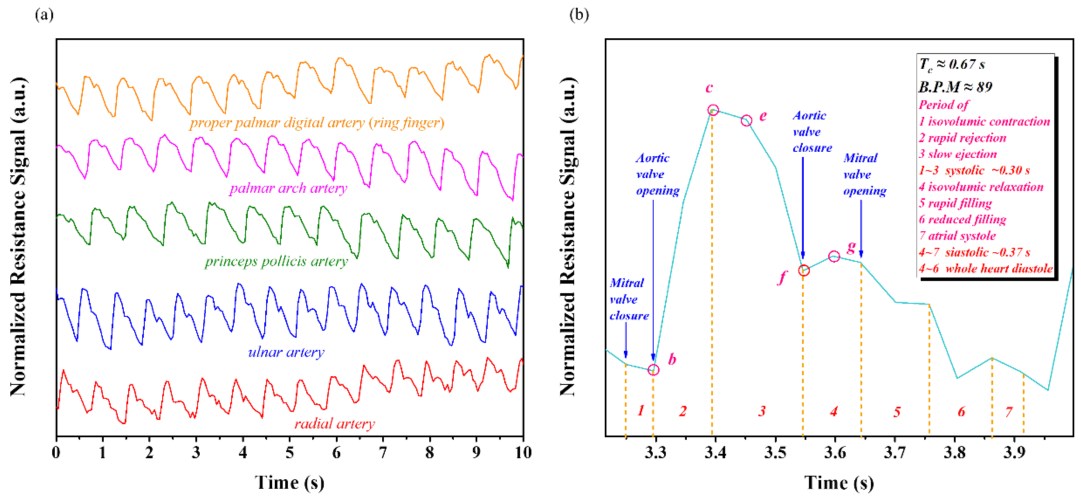

Fig. S5. Normalized resistance signals of arteries. (a) Results of normalized resistance signals of arteries we measured, five artery signals including radial artery, ulnar artery, princeps pollicis artery, palmar arch proper artery and palmar digital artery. (b) Artery signal analysis in a cardiac cycle and more information are given below.

Point **b** : The lowest point of pulse trough corresponds to the position of diastolic pressure monitored by clinical artery. At this time, the left ventricular pressure is equal to the internal pressure of aortic root, which is the beginning of rapid ejection period

Point **c**: The highest point of the whole pulse wave peak corresponds to the position of systolic pressure monitored by the artery clinically. It is generally the first systolic peak of the pulse wave. At this time, the left ventricular pressure is equal to the internal pressure of the aortic root. The rapid ejection period ends and the slow ejection period begins.

Point **e**: It is generally located after the main wave and lower than the peak of the main wave. It is generally the second systolic peak of the pulse wave. The point e is not easy to be observed for people with insufficient blood volume or good arterial elasticity.

Point **f**: The well-know dicrotic notch. At this time, the aorta closes, which is the dividing point between ventricular systole and ventricular diastole.

Point **g**: The point e corresponds to the diastolic peak of cardiac cycle, reflects the reentry wave caused by aortic closure, and is the third systolic peak of pulse wave.

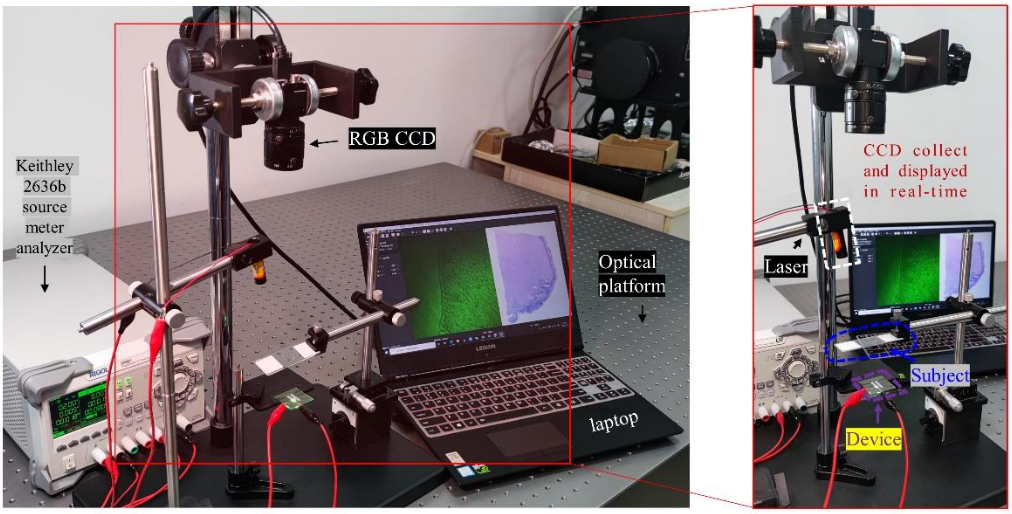

Fig. S6. Actual experiment of imaging.

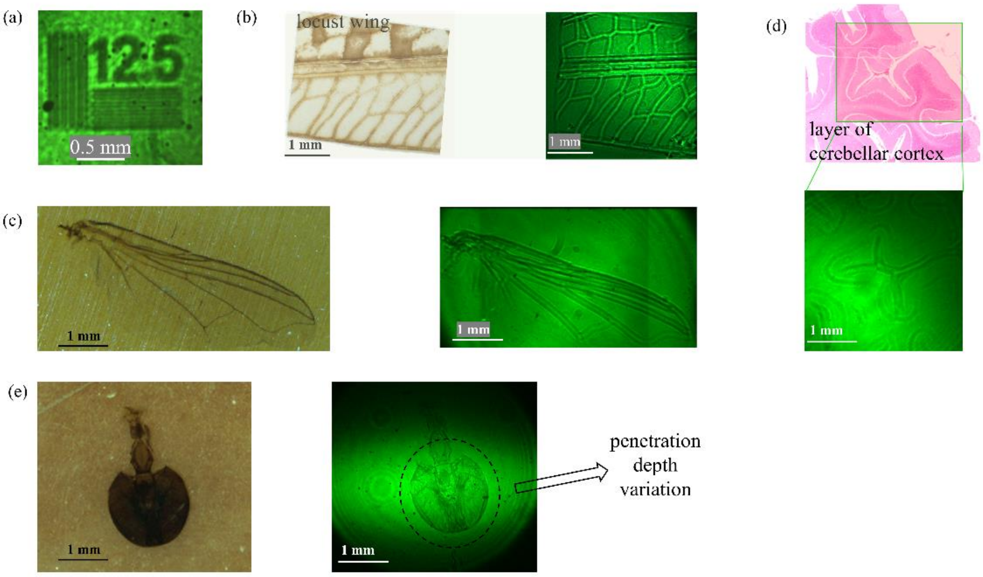

Fig. S7. Resolution test result using a line pair resolution test target and other bio-imaging results in this work. (a) Resolution result with scale of 0.5 mm. (b) The bio-imaging of locust wing with scale of 1 mm. (c) The bio-imaging of fly wing with scale of 1 mm. (d) The bio-imaging of layer of cerebellar cortex. (e) The bio-imaging of housefly mouth with scale of 1 mm.

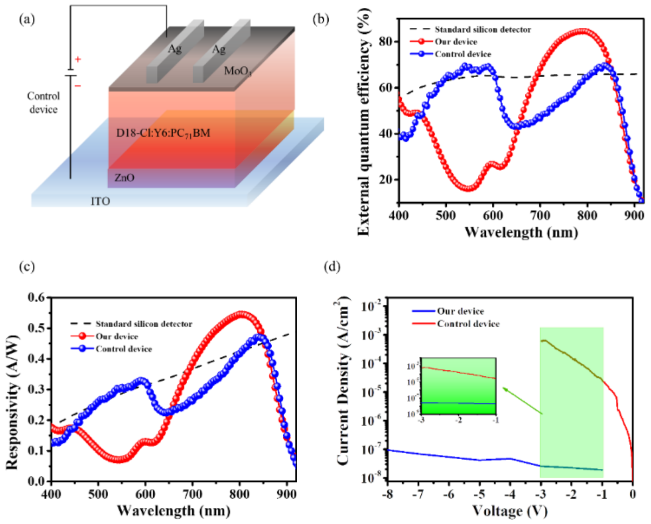

Fig. S8. Structure diagram of contrast device and performance comparison (a) Contrast device with structure of traditional OPD (ITO/ZnO/D18-Cl:Y6:$PC_{71}BM$/MoO_3/Ag). (b) Curves of external quantum efficiency as function of wavelength of our NIR-OPD, constrast device and standard silicon photodetector. (c) Curves of responsivity as function of wavelength of our NIR-OPD, constrast device and standard silicon photodetector. (d) Curves of dark current density with bias voltage of our NIR-OPD and constrast device.

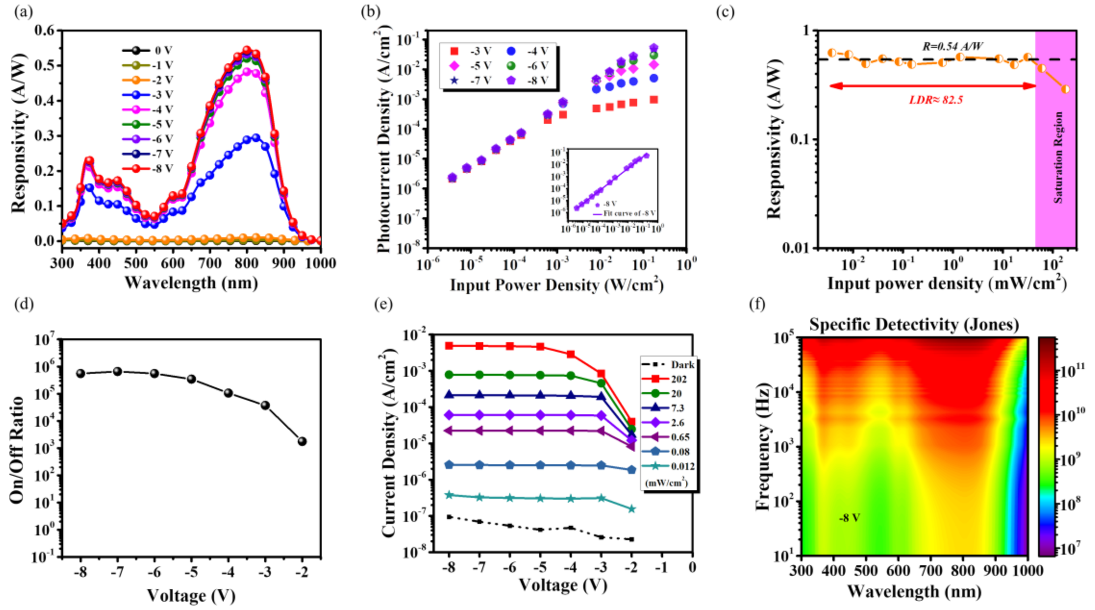

Fig. S9. Detection performance of the NIR-OPD reported in this work. (a) Responsivity as function of applied bias voltages. (b) Photocurrent as function of input power density with different bias voltages applied and liner fit curve at -8 V under 808 nm NIR laser radiation. (c) Responsivity as function of input power density and linear dynamic range at -8 V under 808 nm. (d) On/Off ratio as function of applied bias voltages under 808 nm with input power densinity of 180 mW/cm^2. (e) Photocurrent as function of applied bias voltages with different input power densinity under 940 nm. (f) Specific detectivity mapping as function of both modulation frequency and wavelegth.

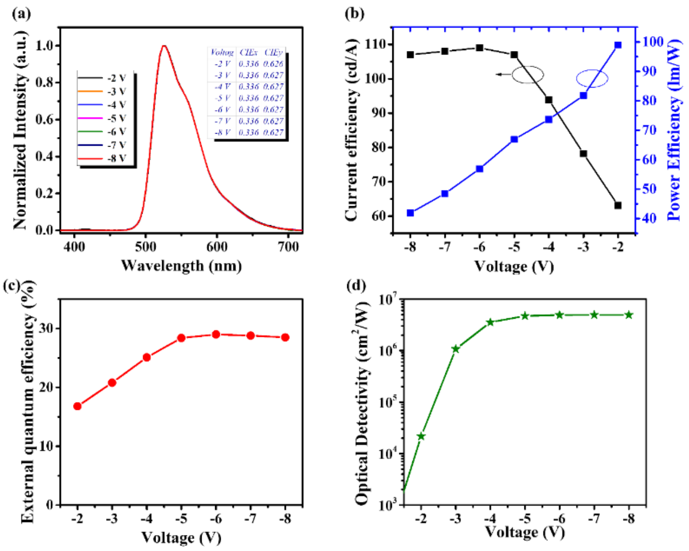

Fig. S10. The characteristics of optical output of the NIR-OPD reported in this work. (a) The EL spectrum of the optical output as function of applied bias voltages and CIE 1931 chromaticity values. (b) The current efficiency and power efficiency of the optical output of the NIR-OPD. (c) The external quantum efficiency of the NIR-OPD. (d) The optical detectivity of the NIR-OPD at input power of 0.7 mw/cm^2 under 850 nm.
